# Analysis of Factors Influencing the Transmembrane Voltage Induced in Filamentous Fungi by Pulsed Electric Fields

**DOI:** 10.3390/microorganisms7090307

**Published:** 2019-09-01

**Authors:** Xuebin Feng, Mengyu Zhu, Jin Xu, Wenqing Yin, Fei Hu

**Affiliations:** College of Engineering, Nanjing Agricultural University, Nanjing 210031, China

**Keywords:** high-voltage pulsed electric field, Filamentous fungi, sterilization, cell membrane transmembrane voltage, nuclear membrane transmembrane voltage

## Abstract

This article studies the sterilization effects of high-voltage pulsed electric field (PEF) of technology on filamentous fungi. A cell dielectric model was proposed based on the physical structure of filamentous fungi. Basic theories of the electromagnetic field were comprehensively applied, and the multiphysics field simulation software COMSOL Multiphysics was used for more detailed study. The effects of PEF treatment parameters and microbial characteristic parameters on the resulting cell membrane and nuclear membrane changes were simulated and analyzed. The results showed significant effects on the transmembrane voltage of the cell membrane and nuclear membrane from the electric field intensity, pulse duration, cell membrane thickness, superposition effect of the pulses. However, the amount of hyphae had little effect, and the number of cell nuclei and the thickness of the cell walls had almost no effect on the transmembrane voltage of the cell membranes and the nuclear membranes. The results provide theoretical support for applying high-voltage PEFs to kill fungi in practical applications.

## 1. Introduction

Pulsed Electric Field (PEF) sterilization technology is an emerging and popular sterilization method in recent years [1]. Its characteristics include low processing temperature, short processing time and low energy consumption. Take into account these characteristics, it plays an irreplaceable role in agricultural engineering, biomedicine and other fields. Contemporary studies have shown that the main effects of PEFs on biological cells are related to local structural changes and destruction of cell membranes. The action of PEF is primarily concentrated on the cell membrane and nuclear membrane. It generates potential differences between the insides and outsides of biofilms, forming transmembrane voltage. When the transmembrane voltage exceeds a certain threshold, it causes physiological changes in the cells and eventually leads to irreversible electroporation of the biofilm to induce apoptosis or cell death [2]. Filamentous fungi are widespread in nature. Take *Rhizoctonia solani* as an example, because of its destructiveness and traditional ways of killing *R. solani* can cause health problems with pesticide residues, it is suitable for PEF treatment. Besides, its hyphae are tubular filaments which can elongate and branch. Many branched hyphae interweave with one further to form mycelium, and the mycelium may contain multiple nuclei.

Overseas studies on high-voltage PEF sterilization began earlier and have been used widely, such as those from the team of Raso from University of Zaragoza [3,4]. Additionally, a mass of studies showed that the transmembrane voltage of cells depends on electric parameters such as field intensity, pulse duration, and the number of pulses. Biological parameters of cells such as cell size and shape also have significant effects [5]. In 1997, an improved Schwan equation was proposed, which was used to calculate the electric field required strength to induce a specific transmembrane voltage in a cell by integrating the Laplace equation:

(1) $$V_{c}=f_{c}E_{c}a\cos\theta$$

(2) $$f_{c}=\frac{l}{l-d/3}$$

In Equations (1) and (2), $V_{c}$ is the breakdown voltage, a is the cell radius, $E_{c}$ is the critical field intensity, and θ is the angle of the given membrane position and the electric field direction. The parameter $f_{c}$ is a shape coefficient related to the length l and the diameter d of spherical or cylindrical cells, and is 1.5 for spherical cells.

These formulae show that the transmembrane voltage is dependent on the field strength and cell size at a given electric field. However, this method does not take the thickness of the cell membranes and cell walls into consideration, and has certain limitations. Beebe et al. [6,7] found that when the pulse duration was small (10–500 ns) and the field strength was high (10–300 kV/cm), electroporation would occur in the organelle membrane such as the nucleus. Elez [8] mainly studied the effects of pulse duration and concluded that a large number of short pulses can kill bacteria more effectively than a small number of long pulses. Maswiwat [9] concentrated on the effects of cellular factors on the transmembrane voltage, without considering the effects of the thickness of cell membranes or the cell walls. Huang et al. at Zhejiang University [10] did a relatively complete simulation study, and considered the influence of cell membrane thickness and cell wall thickness. Their conclusions showed that the sensitivity of microorganisms to PEF treatment was affected not only by electric field parameters but also by biological factors. However, factors such as the number of nuclei and mycelia were not considered. Yao from Chongqing University [11] simulated the inner and outer membrane electroporation model and transmembrane voltage induced by PEF treatments, and fully considered the influence of the nuclei. The disadvantage was that they used human cells as the research object and cell wall thickness was not included in their research scope.

In view of the shortcomings of the previous work, this paper uses electromagnetic field theory to construct a multilayer dielectric model of filamentous fugal cells. The theoretical model is applied to study the effect of PEF on filamentous fugal cells. Additionally, the effects of PEF parameters on the transmembrane voltage of the cell membrane and nuclear membrane were analyzed by exerting the square wave pulse, which is the most widely used at present. These PEF parameters included electric field intensity, pulse duration, the number of pulses, the time between pulses. The filamentous fungal cell parameters were the cell membrane thickness, cell wall thickness, the numbers of nuclei and of mycelia. Therefore, the results of this analysis provide a theoretical basis for high-voltage PEF sterilization.

## 2. Models and Methods

### 2.1. Cell Model

**Figure 1:**
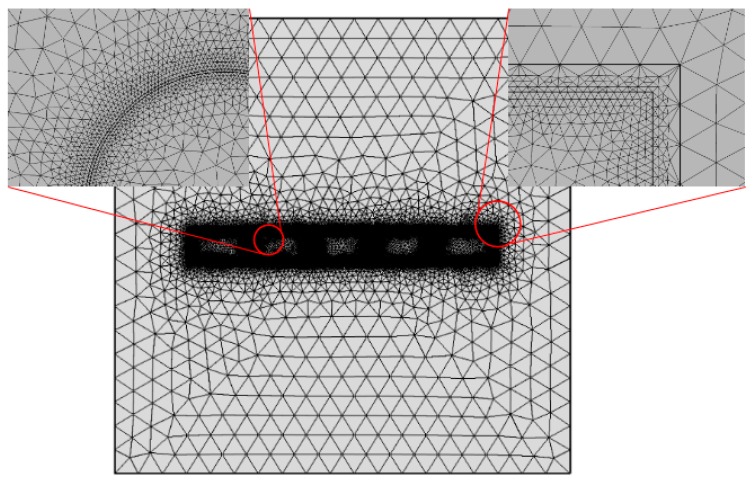
Dielectric model and finite element division of filamentous fungi.

According to the physiological structural characteristics and physical dielectric properties of fungi, each part of a cell can be regarded as an isotropic homogeneous medium. The electrical properties of each part are expressed in terms of conductivity and the relative dielectric constant. In this paper, it was assumed that filamentous fungal cells are slender, and the dielectric model of the filamentous fungal cells was established, as is illustrated in Figure 1. The model is based on *R. solani*, a kind of fungus that consists of a cell wall, cell membrane, cytoplasm, cell nuclear membrane and nuclear cytoplasm. The average hyphae radius is 6–10 μm, and each hyphae usually contains multiple nuclei [12].

In the modeling process of our experiment, since the fungal cells and the surrounding fluid are much smaller than the whole treatment chamber, it can be considered that the fungal cells are within a uniform electric field.

### 2.2. Determination of Simulation Parameters

In this section, the actual electric field is simplified to a uniform electric field. The definition and value of the relevant parameters in the model are derived from typical values for each parameter. The electrical parameters are shown in Table 1 and the geometric parameters are shown in Table 2.

Electroporation of the inner and outer membranes of cells is closely linked to the transmembrane voltage induced by the external PEF. In addition, the parameters such as pulse duration and intensity of the PEF have an essential influence on the perforation efficiency. The electric field parameters studied in this paper include electric field intensity, pulse duration, pulse number and the time interval between pulses. Besides, the same energy but different field intensities and pulse duration are also considered. Additionally, all the pulses used were square waves.

Microorganisms’ structures significantly influence the sterilization effect of high-voltage PEFs. It is necessary to study the structure, size and number of biological cells, and quantify them as their characteristic parameters to influence the cell membrane transmembrane voltage and nuclear membrane transmembrane voltage. Characteristic parameters here refer to the cell size and specific cell parameters for modeling. Additionally, all the characteristic parameters are shown in Table 1 and Table 2. The cell size parameters used in the simulation were: a length of 100 μm, width of 20 μm, cell membrane thickness of 0.005 μm, and a cell wall thickness of 0.02 μm. Additionally, the default cell models were mononuclear and monomycelial.

### 2.3. Simulation Method and Implementation

The current module of COMSOL Multiphysics v5.3a, a professional multiphysics simulation software, was used for the transient analysis. COMSOL Multiphysics is a general engineering simulation software platform with a built-in electromagnetic AC/DC module, which can simulate the distribution and action of an electromagnetic field and give professional simulation results. A five-layer multi-core rod-like dielectric model of fungal cells was established, as it is described in the Section 2.1. cell model. In the simulation model, the entire rod-shaped cell was positioned in an area that was 150 μm by 150 μm, and the electric field intensity was applied through two copper electrodes. Pulse signals were applied at one electrode, and ground through the other. An electrically insulating condition was set at the boundary of the region to make sure that the electric field between the electrodes was a uniform electric field. The mesh subdivision of the simulation model is taken as a more detailed subdivision, and the subdivision unit of the model is 1,000,380 domain units (71,778 boundary units), as is shown in Figure 1.

Since the cell was completely symmetrical in the simulated model, the pressure drop at each point of the cell membrane was the same. Although the position of each nucleus was not exactly the same, considering that the nucleus tends to be near the middle of a cell, the nucleus located in the center-left position was selected to monitor the potential. Based on the above analysis, potential monitoring points were set on the inner and outer surfaces of the cell membrane on the central axis and the inner and outer surface of the nuclear membrane on the left of the central axis.

## 3. Results and Discussion

### 3.1. The Effect of Electric Field Intensity and Pulse Duration

**Figure 2:**
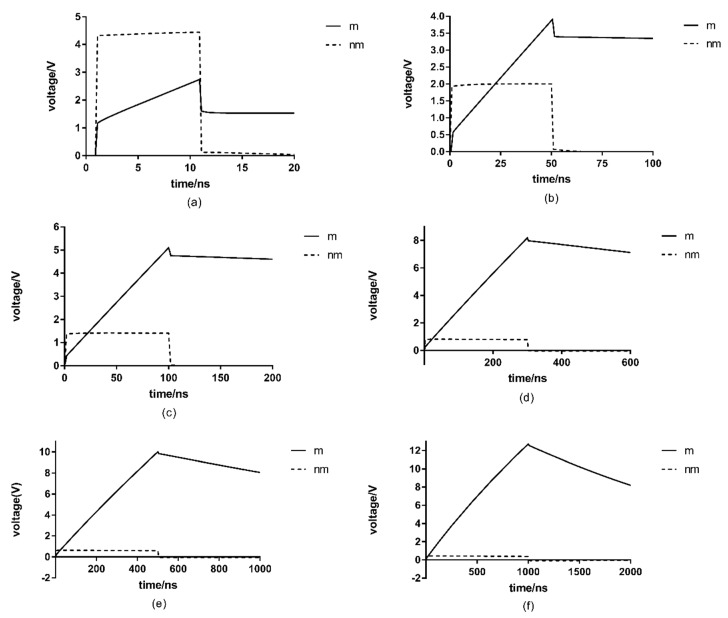
Results from analysis of effects from pulsed electric field on cell membranes and nuclear membranes, for six conditions: (**a**) 130 kV/cm and 10 ns; (**b**) 58 kV/cm and is 50 ns; (**c**) 41 kV/cm and 100 ns; (**d**) 23.8 kV/cm and 300 ns; (**e**) 18.4 kV/cm and 500 ns; (**f**) 13 kV/cm and 1000 ns; m stands for the cell membrane transmembrane voltage and nm stands for nuclear membrane transmembrane voltage.

Electric field intensity is part of the critical parameters that affect the sterilization effect of high-voltage PEF. It is known that the formula for the impulse energy is $\frac{U^{2}T}{RV}$ [11]. U stands for pulse voltage, *T* for treatment time, *R* for equivalent impedance, and V for cell suspension fluid volume. In the simulation model, the pulse energy is kept the same. Six different groups of square wave with field intensities of 130 kV/cm and pulse duration of 10 ns, field intensity of 58 kV/cm and pulse duration of 50 ns, field intensity of 41 kV/cm and pulse duration of 100 ns, field intensity of 23.8 kV/cm and pulse duration of 300 ns, field intensity of 18.4 kV/cm and pulse duration of 500 ns and field intensity of 13 kV/cm and pulse duration of 1000 ns are selected for simulation study on mononuclear and monomycelial cell models. The analysis results are presented in Figure 2.

Depending on the results shown in Figure 2, the cell membrane transmembrane voltage increases linearly and reaches a peak at the end of the duration. Until the pulse disappears, the effective voltage drop on the cell membrane can be maintained for a period of time, and then starts to drop. When the pulse duration increase from 10 ns to 1000 ns, the peak value of cell membrane transmembrane voltage increase from 2.74 V to 12.71 V (maximum value) and the electroporation effect reaches its best.

Cell membrane transmembrane voltage increases with the increasing pulse duration. Even if the electric field intensity is low, when the action time is long enough, higher transmembrane voltage can be obtained on the cell membrane. The electric field parameters of 13 kV/cm field intensity and 1000 ns pulse duration were adopted in the other subsequent studies. The relationship between the transmembrane voltage and the pulse duration obtained by simulation is in consistent with the conclusion reached by Abram [19]. They showed that the degree of inactivation of *Lactobacillus plantarum* increased with longer pulse duration compared to shorter pulse duration. They verified incativation data of *plantarum* and found that the correlation coefficient between the inactivation of *plantarum* and PEF reached 0.879. Basically it suggested that PEF treatment led to the inactivation of *plantarum*.

Nuclear membrane transmembrane voltage differs from that of cell membranes. It reaches peak values in a relatively short time. When the electric field intensity is 130 kV, the peak value of the nuclear membrane transmembrane voltage is close to 4.5 V; while when the electric field intensity is 13 kV, it is less than 0.45 V. The peak value increases with the increase of the electric field intensity and remains basically stable until the applied voltage is removed. The transmembrane voltage generated on the nuclear membrane is highly consistent with the applied excitation waveform. Therefore, the transmembrane voltage of the nuclear membrane is more sensitive to the magnitude of the electric field intensity.

Some studies have shown that when the pulse duration decreases gradually, the electroporation effect gradually transfers from the cell membrane to the organelle membrane [20], which is consistent with our simulation results for the nuclear membrane transmembrane voltage.

### 3.2. The Superposition Effect of Pulses

**Figure 3:**
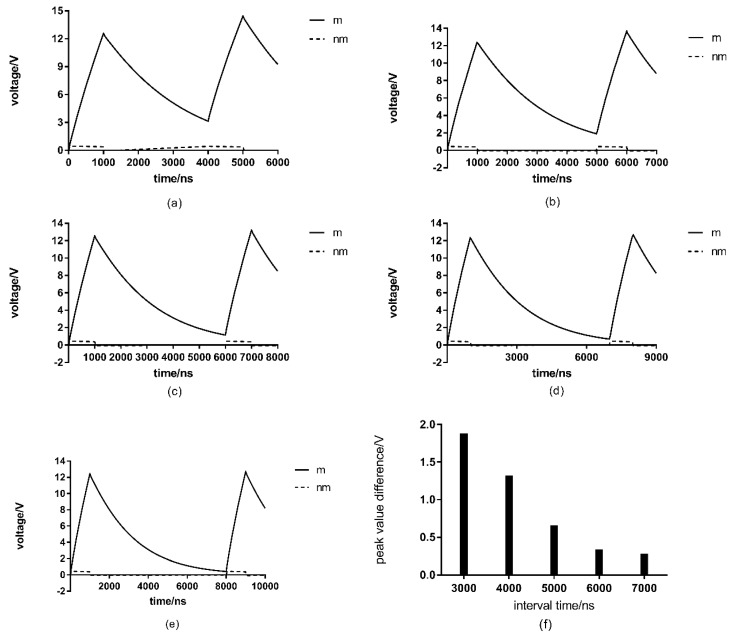
Results of double-pulses at different intervals: (**a**) 3000 ns; (**b**) 4000 ns; (**c**) 5000 ns; (**d**) 6000 ns; (**e**) 7000 ns; (**f**) the difference between the peak cell membrane transmembrane voltage of the first pulse and the peak cell membrane transmembrane voltage of the second pulseat different intervals; m stands for the cell membrane transmembrane voltage and nm stands for nuclear membrane transmembrane voltage.

The number of pulses and the action interval is important parameters that affect the sterilization effect of high-voltage PEF. In this paper, the cumulative effect of multiple pulses within a certain time interval is examined using the simulation model of filamentous fungal cells. Pulses with a field intensity of 13 kV/cm and a pulse duration of 1000 ns were selected, and the double-pulses with an interval of 3000 ns, 4000 ns, 5000 ns, 6000 ns and 7000 ns were also used in this research. The cell models are the same with the former. Additionally, the simulation results are shown in Figure 3.

According to the results shown in Figure 3, pulses have a superposition effect on the transmembrane voltage of the cell membrane. Reducing the interval time between pulses or increasing the number of pulses can cause greater damage to the cell structure of filamentous fungi, giving a better bactericidal effect. With double-pulses with a per-cycle time interval of 6000 ns, the peak value of the transmembrane voltage of the cell membrane reaches 12.33 V at the first pulse and 12.66 V at the second pulse. The difference between the two peaks is 0.33 V, that is, the superposition effect is minimal. With time intervals of 3000 ns, 4000 ns, 5000 ns and 7000 ns, the difference between the two peak transmembrane voltages of the cell membrane are 1.87 V, 1.31 V, 0.65 V and 0.27 V, respectively. It can be possible to conclude that when the time interval is greater than 6000 ns, the superposition effect is no longer significant, thus, 6000 ns is a threshold value. When the pulse duration is greater than or equal to this value, it is of little significance to increase the number of pulses.

As it can be seen from the figure, the transmembrane voltage of the nuclear membrane is relatively small compared with that of the membrane. Additionally, its effect on cells is almost negligible, thus, no detailed analysis will be made here.

According to research by Hülsheger et al. [21], the effects of pulse number can compensate for the effects of pulse duration. That is, when increasing the pulse duration no longer has a significant influence, the sterilizationeffect can be enhanced by increasing the pulse number to make up for the lack of pulse duration, which means that there is a superposition effect. This conclusion is consistent with our research results, but our study also points out that the superposition effect is also limited, the time interval cannot be too long because there is a corresponding threshold. When the interval exceeds this threshold, increasing the number of pulses is meaningless.

### 3.3. The Effect of Cell Membrane Thickness

**Figure 4:**
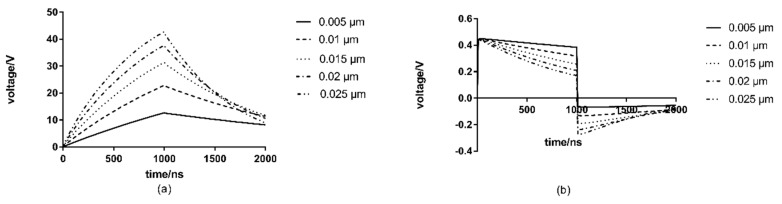
Effect of membrane thickness on transmembrane voltage: (**a**) cell membrane transmembrane voltage (**b**) nuclear membrane transmembrane voltage.

Based on the cell model, the cell membrane thickness is changed to 0.005 μm, 0.01 μm, 0.015 μm, 0.02 μm, 0.025 μm for simulation, and the analysis results are shown in Figure 4.

As the results shown in Figure 4, when the membrane thickness is different, the nuclear transmembrane voltage is significantly different. The transmembrane voltage of the nuclear membrane quickly reaches its maximum value at the beginning, and the maximum value is basically the same for different cell membrane thickness. However, the rate of decrease of the nuclear membrane transmembrane voltage is in contradiction. The rate of decrease of the nuclear membrane transmembrane voltage increases with increased cell membrane thickness.

However, the effect of the cell membrane thickness on the transmembrane voltage is more significant. The cell membrane transmembrane voltage increases with the increased membrane thickness. When the cell membrane thickness is 0.005 μm, the peak potential across the membrane is the lowest, only reaching 12.69 V. However, when the cell membrane thickness is 0.025 μm, the peak value of cell membrane transmembrane voltage reaches 42.96 V. It can be concluded that the cell membrane transmembrane voltage increases significantly with the increase of cell membrane thickness. In other words, the cell membrane thickness is under a significant influence on the cell membranes’ transmembrane voltage.

Huang Kang [10] et al. found that with an increasing cell membrane thickness, the membrane transmembrane voltage only has a slight increase. According to their results, the membrane thickness has a significant impact on the cell membrane transmembrane voltage and the impact is much greater than that in Huang Kang’s studies.

### 3.4. The Effects of Cell Wall Thickness

**Figure 5:**
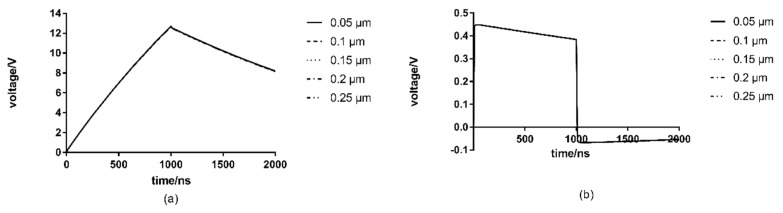
Effects of cell wall thickness on the transmembrane voltage: (**a**) cell membrane transmembrane voltage (**b**) nuclear membrane transmembrane voltage.

Based on the cell model, the cell wall thickness is changed to 0.05 μm, 0.1 μm, 0.15 μm, 0.2 μm, and 0.25 μm for simulation. The analysis results are shown in Figure 5.

Figure 5 shows only two noticeablecurves, indicating that the difference between the simulated results for the cell membrane and nuclear membrane transmembrane voltage with different cell wall thickness is difficult to distinguish, and the data basically coincide. Therefore, biological cell parameters with different cell wall thicknesses have little effect on the cell membrane and nuclear membrane transmembrane voltage. The effect of the cell wall thickness is much less than the effect of the cell membrane thickness.

Combined with the effect of cell membrane thickness, this is inconsistent with the results of Huang et al. [10], who pointed out that the effect of cell wall thickness was greater than that of membrane thickness. However, after a careful comparison, in order to ensure the accurate representation of actual cells, we adopted the current module in the simulation, while Huang adopted the electrostatic module. Different electric field models may also influence the results. In addition, in Huang’s paper, three models were proposed. However, when it came to the influence of the cell wall thickness, only round (the voltage drop was 0.3 V) and oval cells (the voltage drop was 0.207 V) were discussed. While the rod type (which is closest to filamentous fungi) did not give a specific result. Therefore, it is speculated that cell shape has a great influence on the transmembrane voltage. However, the specific reasons need further experimental verification.

### 3.5. The Influence of Nuclei Number

**Figure 6:**
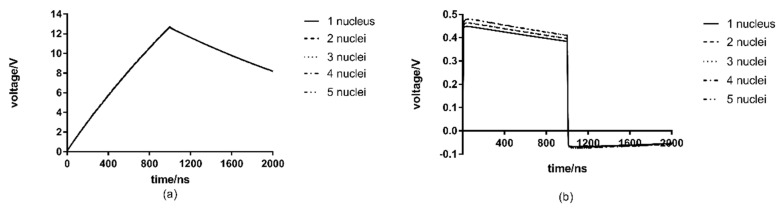
Effects of the number of nuclei on transmembrane voltage: (**a**) cell membrane transmembrane voltage (**b**) nuclear membrane transmembrane voltage.

The number of nuclei in a single fungal cell varies between cells. In this simulation, with all other conditions unchanged, the number of per-cell nucleus in the model is changed to 1, 2, 3, 4 and 5, and then the transmembrane voltage of the cell membrane and nuclear membrane are studied. The nuclei is uniformly distributed in the fungal cells, and are symmetrically distributed in this model. Additionally, the nuclear membrane potential monitoring points are selected from the center or left of the center. The analysis results are presented in Figure 6.

According to the results in Figure 6, the potential across the cell membrane of the tetranuclear cells is the highest (12.77 V) and that of the monocytes is the lowest (12.70 V), and the difference between them is 0.07 V. Additionally, the transmembrane voltage of the binuclear and tetranuclear cells is slightly higher than that of the mononuclear, trinuclear and penta nuclear cells. It is also noteworthy that the transmembrane voltage of cells with odd and even numbers of nuclei increases with increases of the number of nuclei, but the difference is very small.

As for the nuclear membrane, the highest transmembrane voltage at 0.480 V, can be achieved for the nuclear membrane of five-nucleus cells, and the lowest on the mononuclear cells, only 0.448 V, for a difference of 0.032 V and an increase of only 7.14%. Unlike the cell membrane, nuclear membrane transmembrane voltage continues to increase as the number of nuclei increase, regardless of the parity of the number of nuclei. Therefore, the number of nuclei has a different effect on the cell membrane and nuclear membrane transmembrane voltages, and the number of nuclei has a very slight and nearly negligible effect on both.

It can be seen from Table 1 and Table 2 that the electrical conductivity value of the nuclear membrane is very small, namely 1.0 × 10^−3^, and its equivalent impedance is very large, equivalent to an open circuit, which has little impact on the whole model. At the same time, the nuclear membrane is mainly capacitive and far less than the equivalent capacitance of the cell membrane [22]. Therefore, the increase of the number of the nucleus has little effect on the transmembrane voltage of the cell membrane and nuclear membrane.

### 3.6. The Effect of Hyphae Number

**Figure 7:**
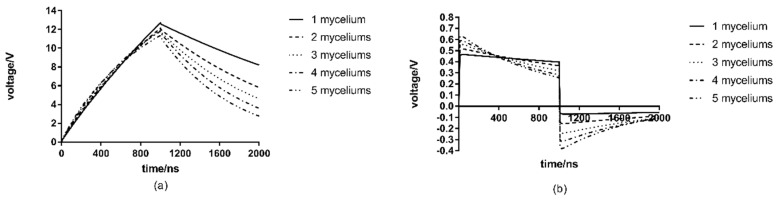
Effects of the number of hyphae on the transmembrane voltage: (**a**) cell membrane transmembrane voltage (**b**) nuclear membrane transmembrane voltage.

Because fungi often group together, there are often multiple hyphae within the range of the same electric field. In order to study the influence of different number of mycelia on membrane and nuclear membrane transmembrane voltage, models with 1, 2, 3, 4 and 5 mycelia are selected in this section. Each hyphae contains three nuclei, and the mycelia is symmetrically distributed above and below the center in the same treatment chamber. The results are shown in Figure 7. The parameters for three nuclei are similar in all mycelia, and there was no overlap between the mycelia. The monitored points were the same as for a single mycelium.

According to Figure 7, compared to the peak value of the cell membrane transmembrane voltage of the single mycelia of 12.70 V, that of five mycelia only reaches 11.34 V, which is less by about 1.36 V. The peak value of the transmembrane voltage decreases with the increase of the number of mycelia, with a maximum decrease of 10.7%.

However, the results are just the opposite comparing to the nuclear membrane. The transmembrane voltage increases with the increase of the number of hyphae. The peak value of the transmembrane voltage with five mycelia is 0.626 V, which is about 0.212 V higher than that of a single mycelia at 0.464 V. The largest increase reached 45.7%. Compared with its effects on the cell membrane transmembrane voltage, the nuclear membrane transmembrane voltage sees a more significant effect from the number of hyphae.

In the actual cell structure of filamentous fungi, mycelia are superimposed, growing and agglomerated, and the number of mycelia is extremely large. It can be inferred that the transmembrane voltage generated by the cell membrane affecting the sterilization effect is far less than the value under the uniform distribution of hyphae in the laboratory in the field experiment of killing typical filamentous fungi such as *R. solani* by PEF, due to the presence of a large number of superposition phenomenon of hyphae. However, the transmembrane voltage of the nuclear membrane is likely to increase further, leading to electroperforation of the nuclear membrane, then causing apoptosis or death, which, of course, needs to be verified by further experiments.

To sum up, from the perspective of electromagnetic field theory, it is of great scientific and application interest to study how cell transmembrane voltage voltage is induced by PEF, to better understanding the electroporation effects of PEF on cells and to guide the use of PEF for killing fungi.

## 4. Conclusions

This paper describes research on the effects found for how the parameters of high-voltage PEF treatment effect the inner and outer membrane transmembrane voltage of filamentous fungi. It also describes how the microorganism characteristics affect those results. According to the analysis, the electric field parameters have significant influence on PEF treatment of filamentous fungi, including pulse duration, electric field intensity; the superposition effect of the pulses. Meanwhile, biological factors also have significant effects, among which the thickness of cell membrane and the number of hyphae have significant effects, while the thickness of cell wall and the number of nuclei have almost no effects. From this research, the following detailed conclusions are drawn:

(1) The electric field intensity and pulse duration have different effects on the membrane and nuclear membrane at the same energy pulse. The electric field intensity has a significant impact on the nuclear membrane transmembrane voltage. The greater the electric field intensity is, the greater the nuclear membrane transmembrane voltage will be. The pulse duration has a great effect on the membrane transmembrane voltage, which obviously increases with increasing of pulse duration. Even at a low field intensity, the membrane transmembrane voltage can reach a considerable value if the pulse duration is long enough. With an electric field of 13 kV/cm, and 1000 ns pulse duration, the membrane transmembrane voltage can reach 12.71 V, while at 130 kV/cm, 10 ns conditions, it can only reach 2.74 V;

(2) When multiple pulses are applied, the cell membrane transmembrane voltage shows an obvious additive effect, but the nuclear membrane transmembrane voltage does not. The additive effect on cell membrane transmembrane voltage is also associated with a pulse interval time. For a pulse cycle interval time of 3000 ns, the peak value of the second pulse can reach 14.52 V, while the peak value can only reach 12.72 V when pulse cycle interval time is increased to 7000 ns;

(3) The cell wall thickness has little effect on cell membrane and nuclear membrane transmembrane voltages. However, the effect of the cell membrane thickness is only reflected in the cell membrane transmembrane voltage, but there it gets a significant effect. When the cell membrane thickness increases from 0.005 μm to 0.025 μm, the transmembrane voltage increases from 12.69 V to 42.96V;

(4) The number of nuclei in a single cell has a little effect on the cell membrane and nuclear membrane transmembrane voltage. The difference between the maximum cell membrane transmembrane voltage and the minimum value, which is 12.70 V, is only 0.07 V;

(5) In contrast to the results with single hyphae, the peak value of membrane transmembrane voltage decreases with multiple hyphae. Compared to the membrane transmembrane voltage of 12.70 V with single hyphae, the membrane transmembrane voltage with five hyphae reaches 11.34 V, about 1.36 V less. The nuclear membrane transmembrane voltage increases from 0.464 V to 0.626 V. The relative positions of the mycelium in the electric field basically do not affect the transmembrane voltages of the cell membranes or nuclear membranes;

(6) The optimal pulsed electric field parameters are electric field of 13 kV/cm, and 1000 ns pulse duration; the number of pulses is as much as possible and the time interval is as short as possible. At the same time, filamentous fungi with thicker cell membrane and fewer mycelia are more likely to achieve better sterilization effect under PEF treatment.

## Figures and Tables

**Table 1:** Fungal cell electrical parameters.

Cell Electrical Parameter	Electrical Conductivity (S/m)	Relative Dielectric Constant
Extracellular fluid	0.1 [10]	90 [13]
Cell wall	0.5 [14]	60 [14]
Cell membrane	1.0E-0.5 [15]	5 [14]
Cytoplasm	1.2 [16]	80 [13]
Nuclear membrane (intima)	1.0E-0.3 [17]	10 [17]
Nucleoplasm	1.0 [17]	80 [17]

**Table 2:** Fungal cell geometric parameters

Geometric Parameters	Value
Hyphae length	100 μm [10]
Hyphae radius	10 μm [18]
Cell membrane thickness	0.005 μm [10]
Cell wall thickness	0.02 μm [10]
Nuclear radius	2 μm NR ^1^
Nuclear membrane thickness	0.04 μm [18]

^1^ NR refers that the data was not valued from reference but determined by devices.
